# NET Rounding: a novel approach to efficient and effective rounds for the modern clinical learning environment

**DOI:** 10.1186/s12909-022-03599-x

**Published:** 2022-08-04

**Authors:** Shirley J. Chan, Hannah L. Archibald, Stephanie M. Conner

**Affiliations:** Department of Medicine, University of California-San Francisco, 521 Parnassus Avenue, Box 0131, San Francisco, California 94143 USA

**Keywords:** Rounding, Efficiency, Patient safety, Clinical education, Resident work hours

## Abstract

**Background:**

Rounds are a foundational practice in patient care and education in the inpatient healthcare environment, but increased demands on inpatient teams have led to dissatisfaction with inefficient, ineffective rounds. In this study, we describe the design, implementation, and evaluation of a novel rounding framework (“NET Rounding”) that provides behaviorally-based strategies to inpatient teams to achieve efficient rounds while preserving patient safety and education.

**Methods:**

NET Rounding consists of nine recommendations divided into three categories: Novel rounding strategies, shared Expectations, and Time management. This framework was introduced as a bundled intervention at a single-site, quaternary-care, academic hospital from March–May 2021. Eighty-three residents and 64 attendings rotated on the inpatient teaching service during the intervention period. Participants were surveyed before, during, and after their rotation about rounding’s contribution to educational value, patient safety, resident duty hour violations and rotation experience. Additionally, rounding duration was recorded daily by team attendings.

**Results:**

Thirty-two residents (38.5%) and 45 attendings (70%) completed post-intervention surveys. Rounding duration was recorded on 529/626 rounding days (80.6%) and resulted in achieving efficient rounds on 412/529 days (77.9%). Residents reported improvement in perceived patient safety (54 to 84%, *p* = 0.0131) and educational value of rounds (38 to 69%, *p* = 0.0213) due to NET Rounding; no change was observed amongst attendings in these areas (79 to 84% and 70 to 80%, *p* = 0.7083 and 0.4237, respectively). Overall, 29/32 residents (91%) and 33/45 attendings (73%) reported a positive impact on rotation experience.

**Conclusions:**

NET Rounding enabled inpatient teaching teams to complete rounds more efficiently while preserving patient safety and education.

## Introduction

In graduate medical education, attending rounds have been a traditional and foundational practice in clinical patient care and medical education in the inpatient hospital setting for over a century [1]. Traditional attending rounds include the attending physician, medical residents, and medical students but in recent decades have evolved to reflect the complexity of the inpatient environment by including advanced practice providers, bedside nurses, patients, and families [2]. The goal of attending rounds has also evolved over time to reflect the diverse priorities of inpatient healthcare delivery, with more emphasis being placed on advancing patient care and updating the morning team with overnight information relative to trainee education and evaluation than in traditional bedside rounds. As a result, both trainees and attendings have reported increased tension and dissatisfaction with rounding practices due to the perceived decrease in clinical education and increased time pressure for task completion [3].

Changes to rounding practices have been attributed to the increasingly demanding and complex inpatient environment, as well as Accreditation Council of Graduate Medical Education (ACGME) work hour restrictions [4]. In fact, studies of rounding length before and after implementation of work hour restrictions have shown progressive lengthening of inpatient rounds from an average of 90–100 minutes before to 122 minutes after, despite decreased time spent on teaching activities and increased perception of time pressure [4, 5]. Most recently, a 2019 time-motion study at our institution found that internal medicine trainees were spending an average of 204 minutes on rounds daily, with only 24 minutes of that time spent on teaching activities [6].

As a result of work hours restrictions and increasing demands for trainee’s time, learning to work efficiently in the medical system has become an important goal for resident education along with traditional topics such as medical knowledge and communication skills [7, 8]. However, many interventions aimed at improving rounding efficiency have focused on reducing time spent on external factors – such as developing structured rounding tools for data gathering, standardizing the electronic medical record (EMR), regionalizing patients, and reducing time-wasting interruptions on rounds – without addressing trainee and attending behaviors during rounds [9–12]. Of note, one study that evaluated the impact of standardizing attending rounds’ structure found that despite reducing rounding length by 8 min, trainees reported decreased satisfaction with rounds and perceived them as lasting 15 min longer [13]. Taken together, these studies demonstrate that simply focusing on reducing time spent rounding is not enough to improve the rounding experience even when efficiency is improved; emphasis must be placed on educational and patient care goals of the inpatient teams as well.

In this study, we describe the design, implementation, and evaluation of NET Rounding: a novel, structured, and goal-oriented rounding strategy aimed at improving rounding efficiency while maintaining the foundational aspects of patient care and clinical education that are critical to inpatient care delivery and trainee education.

## Methods

### Setting and participants

NET Rounding was introduced to the inpatient internal medicine teaching service at a large, urban, quaternary academic hospital in Spring 2021. The teaching service is comprised of eight teams, each with one general internal medicine attending, 1 s or third-year internal medicine resident, two first-year residents, and one to three medical students. Participants included a total of 64 attendings and 83 internal medicine residents across the eight inpatient teams over the course of 3 months (March through May 2021) as previously assigned by their usual clinical schedule (10–14 day rotations for attendings; 2–4 week rotations for interns and residents). Rounds typically (but are not required to) include presentation of overnight admission(s) by a night resident, intern and student “hallway” presentations of established team patients, and bedside education and communication with the patient, family, and multidisciplinary team on most to all established patients. There is a designated start time for rounds, but otherwise no institutional requirement for structure or content of rounds.

### Program description

Using an A3 problem-solving and change management framework, a volunteer working group of 13 internal medicine residents and 10 attendings convened during biweekly meetings over the course of 4 months (September – December 2020) to investigate factors that contributed to ACGME work hour violations on the inpatient medicine service. During analysis of current conditions, the problem of rounding inefficiency was identified and selected by the group for further investigation and intervention.

 As summarized in Table 1, the working group used a 5 Whys framework [14], to perform a root cause analysis for contributors to rounding inefficiency, including structural, cultural, educational and systems-based factors. Next, we generated countermeasures and expected outcomes for every root cause that contributed to rounding inefficiency in order to identify strategies that promote rounding efficiency while maintaining educational value and patient safety. To do this, we interviewed faculty members within the Department of Medicine that were considered expert rounders (“positive outliers”), held group brainstorming sessions, and collected “best practices” from other inpatient services. These findings were synthesized and adapted to create the “NET Rounding” framework.

**Table 1:** Summary of Root Cause Analysis from “5 Why” Exercise, Resultant Countermeasures, and Application to the NET Rounding Framework

Root Cause	Countermeasure	Application to “NET Rounding” Framework
Rounds lack clarity of purpose; purpose of rounds is not shared amongst team members	Define and set expectations amongst team members for rounding agenda, time goals, roles, expectations, and purpose of rounds	Include “Shared Expectations” as a major category for the NET Rounding framework, including: Establishing a daily rounding agenda Identifying goals for rounding
Rounds are structured to accomplish a wide range of tasks in patient care, trainee education, and clinical reasoning	Adopt best practices from other services and positive outliers within Department of Medicine	Apply learned practices to the “Novel Rounding Strategies” and “Time Management” categories, including: Buddy System Rounding Problem Based Planning Rounds Limiting post-presentation comments
Presentations are often unfocused, redundant with information available in the EMR to all team members	Optimize pre-rounding for all team members Pilot alternative rounding methods that better utilize the EMR and time spent on presentations	Gain buy-in to set minimum standards for resident and attending preparation for rounds: Whole team readiness for rounds Prioritizing relevant data and active problems
Efficiency is not valued or taught to residents; “hidden curriculum” epitomizes efficiency as opposite to thoroughness	Educate team members about efficiency mechanisms that can be used during rounds Set time goals to help guide teams in rounds completion	Model prioritization of patient safety and education while ensuring completeness of rounds within a reasonable time-frame: Rounding with purpose Using timers

NET Rounding provides specific strategies to inpatient teams to achieve the goal of completing efficient, effective and educational rounds in 150 minutes or less. NET Rounding is a set of nine rounding strategies divided into three categories (Table 2):

- Novel Rounding Strategies
- Shared Expectations
- Time Management

**Table 2:** NET Rounding strategies organized by category

Category	Rounding Strategy
**Novel** Rounding Strategies	**Whole Team Readiness for Rounds:** All team members commit to coming to rounds with available data from the EMR, thus allowing presentations and discussion to focus on “Assessment and Plan” and not recapitulation of available data
	**Rounding with Purpose:** Team resident will identify a maximum of 6 patients for the team to bedside round on, with emphasis on patients who are critically ill/clinically active, discharging, followed by medical student(s), or have physical exam findings that are essential to clinical decision making and/or are educational. The remainder of patients will be rounded on by “cardflipping” with discussion of active inpatient problems only
	**Buddy System Rounding:** Patient care is to be shared amongst the team rather than being rooted on the intern, with planned accountability for each team member for given task(s). During rounds, the team resident and/or co-intern will utilize a computer during rounds to help complete tasks in real time. Team attending and/or resident should identify tasks on rounds that they will complete independently to offload the intern’s post-rounds tasklist
	**Problem-Based Planning Rounds:** Presenters will pause after each system/problem for the team to discuss and finalize a plan for that problem before moving on to the next problem. The goal of this is to minimize the “stacking” of feedback and summarizing the plan that can occur at the end of patient presentations
Shared **Expectations**	**Establishing a Daily Rounding Agenda:** Team leader (senior resident and/or attending) will establish a specific and time-based agenda for rounds (including holdover signout), and communicate that agenda with time goals to the team before the start of rounds
	**Identifying Goals for Rounding:** The team will identify specific and measurable goals for rounding efficiency and task delegation at the start of rotation, with planned evaluations for all team members at mid-point and end-of- rotation feedback
**Time** Management	**Using Timers:** Team leader (senior resident and/or attending) will utilize a timer for student and intern presentations that progressively shortens presentation length to the goal average of 7 minutes per established patient and 12 minutes per new patient.
	**Prioritizing Relevant Data and Active Problems:** Interns and residents will present relevant data, assessments and plans for active problems only (usually 2–3); stable/chronic problems should either not be verbally discussed if no change/updates are available, or discussed in one brief statement.
	**Limiting Post-Presentation Comments:** Limit feedback to one team member per presentation and/or reschedule presentation related, individualized feedback either to transitioning between patients or to after rounds. Avoid reiterating plans.

Inpatient teams were asked to choose at least one rounding strategy from each of the three categories and to implement this bundled intervention for the duration of their rotation (2 to 4 weeks).

Prior to beginning the intervention, residents and attendings were educated about NET Rounding rationale, key principles, and strategies through e-mail announcements, placards in common spaces, and didactic sessions for both residents and attendings. Pocket cards were created and disseminated for reference. During the intervention, volunteer resident and attendings were available to address questions and barriers for teams implementing NET Rounding. Inpatient teams were incentivized with nominal gift cards for team-based participation and individual survey completion. Of note, medical students were excluded from this intervention.

### Program evaluation

NET Rounding was assessed using online surveys disseminated via e-mail to team residents and attendings before and after their scheduled rotation, a daily rounding report tool, and available team patient census data. The primary outcome was rounding duration; secondary outcomes included the impact of rounding on patient safety, educational value, resident perception of work hour violations, and overall rotation experience. These outcomes were chosen to specifically address the primary driver behind the A3 problem-solving method (rounding inefficiency as a contributor to resident work hour violations), while also considering the traditional goals of rounds themselves (patient care, trainee education) and provider experience of team members during the pilot.

Baseline data for rounding duration and perception of rounding impact on educational value, patient safety, and resident duty hour violations were collected via a five question online survey pre-intervention. During the intervention, a brief three question rounding report tool was disseminated daily to team attendings to track rounding duration and patient safety events. Post-intervention, we assessed NET Rounding impact on rounding duration, perception of educational value, patient safety, resident duty hour violations and rotation experience using a 13 question online survey. A 5-point Likert scale was used for responses in all assessments, except for patient safety event reporting which was recorded as a binary yes/no response with optional free text for further description. Additionally, resident duty hour violations were de-identified and collected from a user-restricted, institutional work hours dashboard for the 4 months pre-intervention (November 2020 – February 2021) and during the intervention for comparison [15]. Patient census by team over the intervention period was tracked as a possible confounding variable. Informed consent, study design and survey documents were submitted to the IRB and received approval via quality improvement designation. Chi-square analysis was used to compare pre- and post-intervention survey data. Resident work hour violations and team patient census data over the intervention period was compared using unpaired t-tests for independent samples and Analysis of Variance analysis (ANOVA), respectively.

## Results

Among the 83 residents who rotated on the inpatient teaching service during the intervention period, 39 (47%) completed the pre-intervention survey and 32 (38.5%) completed the post-intervention survey. Among the 64 attendings who rotated, 43 (67%) completed the pre-intervention survey and 45 (70%) completed the post-intervention survey. Daily report of rounding duration and patient safety events was completed by attendings on 529 of 626 possible rounding days during the intervention (81%).

**Fig. 1:**
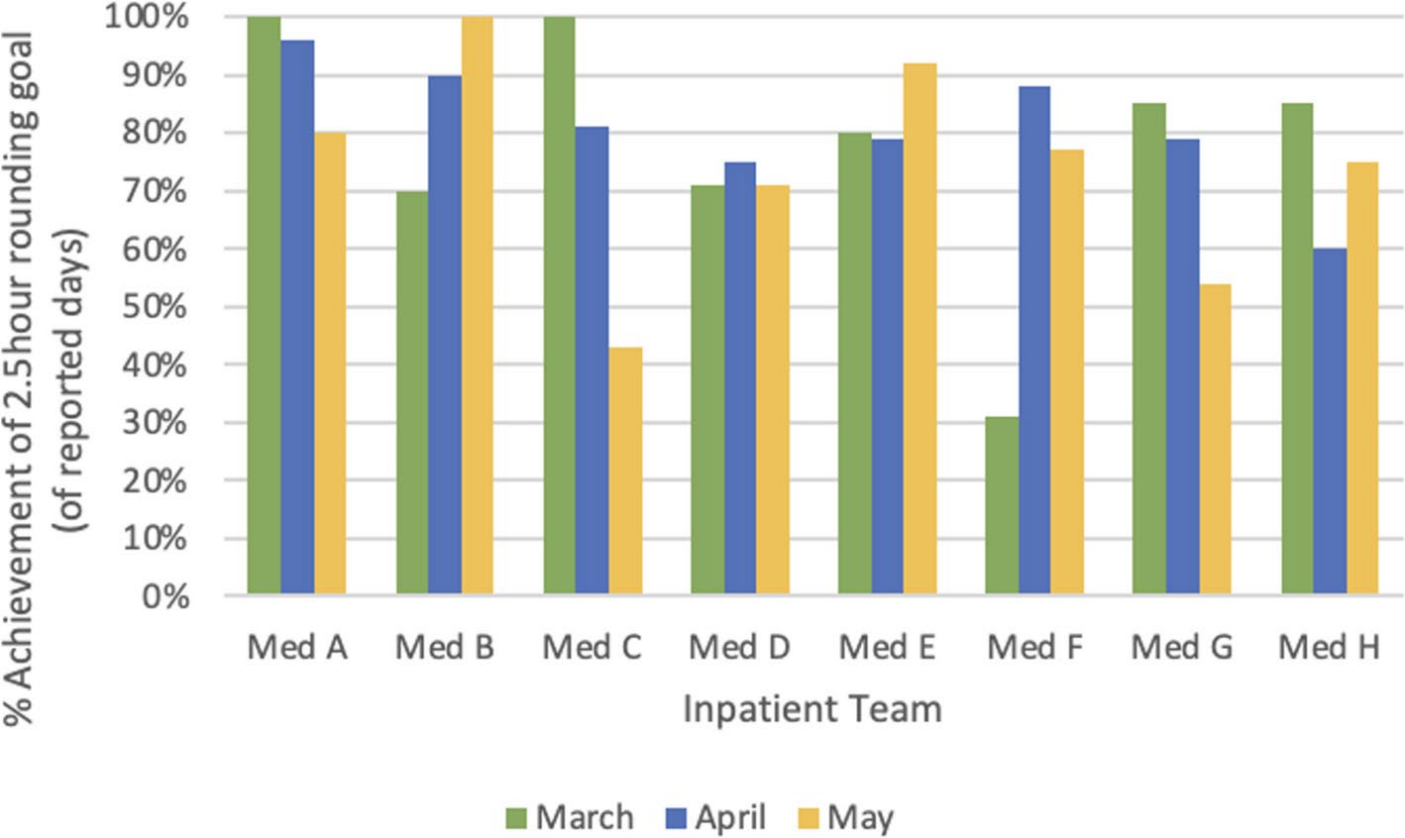
Percent achievement of daily rounding time goal by team and month

Both groups reported high rates of NET Rounding implementation, with 24 (75%) resident respondents and 33 (73%) attending respondents reporting daily use of NET Rounding strategies during their rotation. After implementing NET Rounding strategies, rounds were completed within 150 minutes or less on 412 of 529 measured rounding days (78%) across the eight inpatient teams (Fig. 1). Additionally, the average team patient census was not significantly different over the 3 months of the intervention period: 9.2 ± 1.3 patients in March, 9.3 ± 0.8 in April, and 8.7 ± 0.6 in May (*p* = 0.498, ANOVA).

Among the nine NET Rounding strategies that teams could choose from, four were identified by 68 of 74 attending and resident respondents (92%) to be most helpful in achieving rounds efficiency:

1. Rounding with Purpose (20/74, 27%)
2. Whole Team Readiness for Rounds (18/74, 24%)
3. Prioritizing Relevant Data and Problems (15/74, 20%)
4. Establishing a Daily Rounding Agenda (15/74, 20%)

Post-intervention, both groups reported achieving the target rounding duration of 150 minutes or less more than 80% of the time; significantly more than pre-intervention (Table 3). Additionally, residents reported that NET Rounding significantly improved their perception of patient safety and educational value of rounds. However, there was no similar effect perceived by attendings, though notably they reported a higher baseline score for both of these outcomes pre-intervention.

**Table 3:** Resident and attending perceptions of average rounding duration, patient safety, educational value, and self-reported work hour violations before and after NET Rounding

Outcomes	Residents			Attendings		
	Pre (*n* = 39)	Post (*n* = 32)	***p***-value	Pre (*n* = 43)	Post (*n* = 44)	***p***-value
*Average rounding length*						
< 150 minutes	8	29	**< 0.0001**	7	37	**< 0.0001**
> 150 minutes	31	3		36	8	
*Frequency of safe plan by end of rounds*						
Almost always	21	27	**0.0131**	34	38	0.7083
Other response	18	5		9	7	
*Educational value of rounds*						
Very – extremely valuable	15	22	**0.0213**	30	35	0.4237
No – moderately valuable	24	10		13	9	
*Resident report of work hour violations*						
Never	11	25	**< 0.0001**	–	–	**–**
Other response	28	7		–	–	

No patient safety events were reported by residents due to NET Rounding, though one attending reported a near-miss safety event related to incomplete discharge medication reconciliation due to abbreviated rounding (1/529, 0.19%). Additionally, no attendings or residents reported that decreased length of rounds resulted in decreased quality of education on the inpatient teaching service, though both residents and attendings noted a shift from teaching/learning predominantly occurring on rounds to being more evenly distributed between on/off rounds.

Resident perception of work hour violations significantly decreased after NET Rounding (Table 3). Attendings agreed, with 19 (42%) identifying fewer work hour violations amongst residents as a benefit of NET Rounding. However, both the baseline perception of work hour violation frequency and the impact of NET Rounding was found to be overestimated based on available data from the work hours dashboard, which showed a modest, non-significant decrease in work hour violations pre-intervention compared to post (4 to 2.6%, *p* = 0.235, t-test). Additionally, attending self-report of their own “work hours” decreased by a median of 1 h, from 10 to 9 h daily.

Overall, 29 resident respondents (91%) and 33 attending respondents (73%) reported NET rounding having a positive or very positive impact on their rotation experience. The most frequently reported benefits included faster patient care delivery (57/74, 77%), earlier task completion (43/74, 58%), and increased time spent with patients and families after rounds (36/74, 49%). Only seven attending and resident respondents (9.1%) did not experience any benefits to NET Rounding. In contrast, 35 (45.5%) did not experience any barriers to NET Rounding implementation. Few barriers to implementation were reported, but most frequently included high team census (12/74, 16%), high patient acuity/complexity (11/74, 15%), and perception that efficient rounds were being achieved without NET Rounding strategies (9/74, 12%).

## Discussion

Achieving efficient, safe and educational rounds can be challenging in the modern-day clinical learning environment due to the complexities of navigating safe patient care, trainee educational needs, health system priorities, and ACGME work hour restrictions. However, the NET Rounding framework enabled teaching teams at a large quaternary academic hospital to complete rounds more efficiently while preserving educational value, patient safety, and work hour adherence. Moreover, NET Rounding was readily implemented by inpatient teaching teams, improved the rotation experience for residents and attendings, and had few barriers to implementation. By engaging attendings and residents in this shared rounding framework, NET Rounding offers significant benefits to workflow efficiency, patient care, residency education, and team satisfaction.

Through NET Rounding, we demonstrated that implementing a bundled intervention with emphasis on improving individual efficiency behaviors, creating shared expectations amongst team members, and establishing time-based goals can effectively shorten the length of daily attending rounds while improving the value and experience of rounds for team members, particularly trainees. We assessed rounding length continuously throughout the intervention using the daily rounding report tool, in addition to post-rotation survey, in order to minimize the impact of recall bias on daily rounding length. We also tracked patient census data over the intervention period to account for this potential confounder on rounding length; no significant difference in patient census was seen. Additionally, the resident-reported decrease in work hour violations as well as decreased attending median work hours suggests that decreased rounding length contributed to shorter workdays for all team members and did not simply shunt rounding activities into off-rounding times.

Attendings and residents had differing opinions on the patient safety contribution and educational value of rounds before the NET Rounding intervention, and NET Rounding served to positively impact the residents’ rounding experience more than attendings. Prior to the intervention, attendings had a higher baseline perception of patient safety by the end of rounds in comparison to residents (79% vs 54%). Attendings also had a higher baseline perception of the educational value of rounds compared to residents (70% vs 38%). This supports one of the main premises suggested by our initial root cause analysis: that residents and attendings have different a priori views regarding the value of rounds and how well rounds are meeting their goals, which is likely related to their respective roles in ensuring safe patient care and contributing to the clinical learning environment. Furthermore, this likely reflects the outsize importance that attendings place on rounds as the dominant clinical and educational activity with the team; in contrast, residents work alongside each other for most of the day, yielding more opportunity outside of rounds for clinical decision making and education. We were encouraged to find that the NET Rounding intervention preserved attendings’ positive perceptions of patient safety and educational value, while significantly improving these domains for residents. This finding underscores the importance of creating a shared rounding framework between residents and attendings in order to maximize the efficiency, effectiveness, and educational value of rounds for both groups.

Interestingly, the vast majority of attendings and residents (92%) found four of the available nine rounding interventions to be most effective: Rounding with Purpose, Whole Team Readiness for Rounds, Prioritizing Active Data and Problems, and Setting a Daily Rounding Agenda. While detailed characterization of this requires further qualitative analysis that is forthcoming, we speculate that these four strategies were favored for two main reasons. First, all four were adapted from individual rounding strategies reported by positive outliers within the Department of Medicine, not from external departments or experiences from other residency programs (as was the case with both Buddy System Rounding and Problem Based Planning Rounds). It is likely that versions of those rounding strategies were being used with some frequency by members of the inpatient team prior to the intervention (though not defined or coupled with other elements of the NET Rounding intervention). Actively naming the importance of these strategies and their familiarity reduced the activation energy to try them and use them with success. Second, all four interventions clarified variable elements of the “hidden curriculum” that exists in the rounding culture, including clarifying intern and resident expectation(s) of attendings (Whole Team Readiness for Rounds), attending and intern expectation of resident rounds leadership (Rounding with Purpose, Setting a Daily Rounding Agenda) and attending and resident expectation of intern presentations (Prioritizing Active Data and Problems). Furthermore, reducing uncertainty about the structure, timing, and individual roles within rounds, while promoting individual autonomy within the group is known to have a positive effect on team performance and morale [16].

There were a few limitations to note in the implementation and evaluation of NET Rounding. First, we implemented this framework at a single site with “traditional” inpatient teaching teams, which limits generalizability to other practice settings or multidisciplinary teams. Second, the intervention period occurred later in the academic year, when residents and interns are relatively experienced and will likely encounter fewer barriers to implementation within their established rounding practices. However, while we expect that some aspects of NET Rounding may be more difficult for early-year trainees to adopt (particularly some of the novel rounding strategies), many of the NET Rounding principles involving shared expectations and time management can be incorporated regardless of learner level. Third, the response rate from residents was lower than attendings and did not achieve 50%, which can serve to skew results from this group. Lastly, we did not directly measure the impact of NET Rounding on medical student or patient experience as this was deemed out of the scope of this particular study, though we are encouraged that the reported benefits of NET Rounding would seemingly benefit these groups as well. We aim to specifically address these limitations in future studies.

## Conclusions

NET Rounding is a novel framework that can be used to guide residents and attendings toward efficient, effective, and educational rounds within the inpatient clinical learning environment. This study demonstrates that creating a shared framework amongst team members for the purpose and practice of rounds can achieve rounding efficiency while maintaining the foundational aspects of patient care and clinical education that are critical to inpatient care delivery and resident education.

## Data Availability

The datasets used and/or analysed during the current study are available from the corresponding author on reasonable request.

## Abbreviations

ACGME: Accreditation Council of Graduate Medical Education
EMR: Electronic Medical Record
ANOVA: Analysis of Variance Analysis
